# Nephrology in Nepal: How it Started and Where We Are

**DOI:** 10.34067/KID.0000000991

**Published:** 2025-09-03

**Authors:** Deepak Sharma, Taranath Sharma, Jie Tang

**Affiliations:** 1 Department of Internal Medicine and Nephrology, Dhulikhel Hospital, Kathmandu University School of Medical Science, Dhulikhel, Nepal; 2 Department of Internal Medicine, College of Medical sciences, Kathmandu University, Bharatpur, Chitwan, Nepal; 3 Division of Kidney Diseases and Hypertension, Alpert Medical School of Brown University, Providence, Rhode Island

**Keywords:** clinical nephrology, chronic kidney disease, clinical nephrology, dialysis, dialysis access, ESKD, health equity, diversity, and inclusion, health policy, health status, nephrology, renal transplantation

## Abstract

Nepal has made significant strides in nephrology, progressing from no dialysis services to offering comprehensive kidney care, including dialysis and transplantation. The journey began in the 1980s with the establishment of the first nephrology unit at Bir Hospital and has since grown to encompass over 60 dialysis centers and multiple transplant facilities. Government initiatives, such as financial subsidies and health insurance schemes, have increased accessibility, yet disparities persist due to workforce shortages, uneven infrastructure distribution, and inconsistent quality control in dialysis services. Predialysis care remains underdeveloped, and challenges in adopting peritoneal dialysis persist due to socioeconomic and logistical barriers. The kidney transplant program, primarily reliant on living donors, has shown steady growth. Critical care nephrology and interventional services are gradually emerging. To advance, addressing workforce shortages, improving care quality, and expanding equitable access through strategic investments and global partnerships are essential.

## Introduction

**Figure 1:**
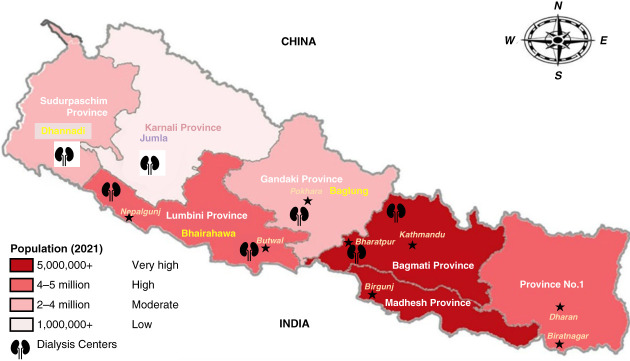
Map of Nepal with population distribution and nephrology facility and dialysis centers.

Nepal, soon going to approach the milestone of graduating from the list of least developed countries,^1^ is a landlocked country situated between India and China in South Asia, popularly known as a yam between two boulders' as King Prithvi Narayan Shah described over 250 years back. Nepal covers an area of 147,516 km^2^, is home to Mt. Everest, and is the birthplace of Gautam Buddha with a present-day population of 29,622,697 (Figure 1).^2^

## Health Care Delivery

The health care delivery follows a hybrid approach with public, private, and community-based nongovernment organizations providing health services in alignment with national policies defined by the Ministry of Health. On October 10, 2017, Nepal's legislative body approved the National Health Insurance bill, which provides coverage of 100,000 rupees per year for a family of five (approx. 725 USD per 2025 exchange rate), and is accepted at most public and private facilities. However, the annual premium of 3500 rupees (26 USD) and 20% co-pay may seem burdensome for most of the population. The government insurance bill provides patients with CKD, whether undergoing dialysis or post renal transplant, with 5000 rupees (36 USD) per month for free hemodialysis twice weekly, peritoneal dialysis (PD) fluid supply, or medications. The bill also includes a program that covers the cost of a kidney transplant up to 550,000 rupees (3979 USD) if the surgery is performed at the government facilities. However, owing to a shortage of dialysis centers and government-sponsored transplant facilities, most of patients are unable to access appropriate KRTs. To address the disparity and improve access, the Nepal government has begun agreements with private facilities to provide KRT and waiving premium payments for individuals living in severe poverty directly to the service provider. The government also provides a subsidy through the Disadvantaged Citizens Medical Treatment Fund to any citizen unable to afford for their health care costs and diagnosed with one of eight eligible chronic illnesses: cardiovascular disease, cancer, renal failure, Alzheimer disease, Parkinson disease, head and spinal cord injury, sickle cell anemia, or stroke. To receive subsidized treatment for these chronic conditions, patients must obtain the necessary medical documents from their local government health authorities. This subsidized care is only available in certain government-approved health care sites. By contrast, wealthy patients often seek treatments in the private sector. Nepal's largest cities, including Kathmandu, Pokhara, Bharatpur, Dharan, Butwal, Biratnagar, Birgunj, and Nepalgunj, are home to both public and private hospitals that offer excellent medical care (Table 1).

**Table 1:** Current status of nephrology services in Nepal (2025)

Parameter	Value	Source
Population of Nepal	Approximately 30 million	Census Nepal, 2021
Practicing nephrologists	Approximately 56	Nepal Society of Nephrology, 2023
Dialysis centers	>70 (public and private)	Nepal Kidney Foundation, 2023
Hemodialysis machines	Approximately 600	NKF, author data
Patients on maintenance hemodialysis	Approximately 4000	NKF, author data
PD utilization	Approximately 11% of ESKD patients	Author data
AVF use at dialysis initiation	Approximately 25%	Basnet *et al.*^13^, Nepal Med J 2021
Government dialysis subsidy	2500 NPR (approximately 18 USD) per session	MoHP
Transplant centers (Govt+Pvt)	Approximately 8	MoHP, SDNTC, TUTH, author data
Pediatric transplant availability	Limited, <10% of total	Kafle *et al.*^22^, Pediatr transplant 2019
Insurance for kidney care	Available through NHI and chronic disease fund	Health Insurance Board, Nepal 2023

AVF, arterio-venous fistula; MoHP, Ministry of Health and Population; NHI, national health insurance; NKF, National Kidney Foundation; NPR, Nepalese rupee; PD, peritoneal dialysis; SDNTC, Shahid Dharmabhakta National Transplant Center; TUTH, Tribhuvan university teaching hospital.

## Journey of Nephrology in Nepal

Nepal's oldest hospital, “Bir Hospital,” founded the country's first nephrology department in the 1980s to meet the growing demand for dedicated renal care. This was led by the late Dr. Puskar Raj Satyal, a pioneer of Nephrology in Nepal, and was later joined by Drs. Rishi Kumar Kafle and Sudha Khakurel. Nepal lacked dialysis facilities until the early 1980s when Bir Hospital in Kathmandu started providing intermittent PD. The first hemodialysis unit was established in the same hospital in 1986, with the support from Indian government, and initially operated with two hemodialysis machines. A second dialysis unit was opened in the Tribhuvan University Teaching Hospital (TUTH) in 1996, followed by a few more dialysis facilities affiliated with private hospitals around the Kathmandu Valley and outside the capital. The National Kidney Center was established in 1997 funded by Nepal Kidney Foundation with the aim of providing free or subsidized dialysis to most Nepalese ESKD population. Dr. David Francis, a transplant surgeon from Australia along with a group of Nepalese surgeons and nephrologists led by Dr. Dibya Singh Shah, performed the first successful renal transplant at TUTH in August 2008.^3^ The Shahid Dharmabhakta National Transplant Center (SDNTC) was later established by the Ministry of Health in 2012 to advance organ transplantation in Nepal (Tables 1-3).

**Table 2:** Milestone of nephrology care in Nepal

Yr/Period	Key Developments
1960s	Limited awareness; basic medical facilities in major hospitals
1980s	Introduction of dialysis; initial kidney disease awareness
1990s	Formation of Nepal Kidney Foundation (1993); training programs; dialysis units established in major hospitals
2000s	Expansion of dialysis centers; nephrology recognized as a specialty; early kidney transplantation programs begin
2010s	Increased trained nephrologists; national guidelines for CKD and dialysis; improved awareness and management
2020s	Modernized kidney care services; expanded transplantation programs; ongoing research and prevention strategies

**Table 3:** Kidney transplantation in Nepal (2008–2025)

Yr	Key Milestones
2008	First successful living donor transplant (TUTH)
2012	SDNTC established by Ministry of Health
2015	Government funding for transplantation launched (up to 550,000 NPR per transplant)
2016	First brain-dead donor transplant (SDNTC)
2017–2020	Steady increase in transplant volume across government and private centers
2021–2025	Expansion to Pokhara Academy of Health Sciences and other regional centers
Cumulative (as of 2025)	∼3090 transplants performed (majority living related; small number of brain-dead donors)

NPR, Nepalese rupee; SDNTC, Shahid Dharmabhakta National Transplant Center; TUTH, Tribhuvan University Teaching Hospital.

Financial incentives for dialysis treatment were started in 2011. Hemodialysis centers received 97 USD (approx. 8500 rupees as per 2011 exchange rate) per patient per year for the services provided, which was later increased to 483 USD (approx. 41,500 rupees) in 2012. As a result of financial incentives, the number of dialysis centers increased dramatically after 2010, reaching 42 in 2016 and 60 in 2021, with 570 hemodialysis machines and about 3775 patients receiving dialysis during those years.^4,5^ In 2014, a free hemodialysis campaign was launched, allowing twice-weekly sessions for a year, which was later expanded to a twice-weekly service for 2 years in 2015. In the same year, the funding was expanded to cover kidney transplantation with the initial reimbursement capped at 550,000 rupees (approx. 5340 USD as per 2015 exchange rate) in total and then 5000 rupees (49 USD) every month afterward. In 2016, the Ministry of Health started free lifetime hemodialysis in Nepal which is provided by all public hospitals and a few selected private facilities contracted with the ministry. Currently, dialysis centers receive 2500 rupees (18 USD) per session of hemodialysis provided to the patient. To support PD, 90 bags of free PD dialysate were provided by the government each month to facilities providing continuous ambulatory peritoneal dialysis.^4^

Free dialysis was started in Nepal primarily as a public health initiative to address the growing burden of CKD and ESKD, particularly among economically disadvantaged populations. Nephrologists, health professionals, and patient advocacy groups played a major role in bringing attention to kidney disease as a public health crisis and lobbying for free dialysis as there was immediate concern regarding the increased incidence of CKD and ESKD in Nepal due to risk factors such as diabetes, hypertension GN, and environmental causes.^6^ The need for maintenance dialysis, especially hemodialysis, was growing rapidly without adequate financial support mechanisms. As dialysis is expensive and lifelong, making it unaffordable for most patients in Nepal, before the government program, many patients discontinued treatment or died prematurely due to financial constraints. With constant effort of advocates, the Government of Nepal included free dialysis services under its commitment to universal health coverage and right to health, as enshrined in the 2015 Constitution of Nepal which aligns with sustainable development goals, particularly Goal 3: “Ensure healthy lives and promote well-being for all at all ages.”^7^ In 2016, Ministry of Health and Population officially began the free hemodialysis program which included free dialysis at designated public and private hospitals. Patients are provided up to two sessions per week (later extended in special cases) and kidney transplant patients are also supported with pretransplant and post-transplant dialysis. These steps aimed to prevent catastrophic health expenditures and medical impoverishment (Table 1).

## Present Day Nephrology in Nepal

### Work Force

Despite the high demand, the nephrology work force has been limited. The ratio of patient-to-doctor is wildly out of proportion. Currently, only 56 nephrologists are practicing in the country, serving just 15% of the population.^8^ Most adult nephrologists are practicing in Kathmandu, the capital city. Sixty percent of physicians work in the private sector, which is already saturated and provides up to two thirds of the country's capacity. Many doctors engage in “dual practice,” working in both the public and private sectors. This can lead to conflicts of interest, reduced quality of care in the public sector, and increased health care costs.^9^ Furthermore, there has been a severe shortage of trained dialysis nurse and ancillary staff including technician and renal dietician, which limits the effective delivery of service.

## Predialysis CKD Care

The predominant causes of CKD in Nepal are hypertension, diabetes, and other cardiometabolic risk factors, with glomerular diseases being important in children. Occupational and environmental exposures, including heat stress, dehydration, nonsteroidal anti-inflammatory drug use, and possible toxin exposure, may play a contributory role in certain populations, but the overall burden remains primarily driven by traditional risk factors.^6,10,11^ A epidemiologic study reported prevalence of CKD to be 6.0% in Nepal.^6^ The actual prevalence is likely much higher due to the lack of disease screening. To address the rising incidence of CKD, a cohesive kidney care strategy is essential. This strategy should focus on effective prevention, early detection, and optimized care to delay CKD progression. Primary care physicians (PCPs) and nephrologists should collaborate closely, with PCPs playing a key role in identifying and managing early-stage CKD and appropriately referring patients with CKD to establish care with nephrologists. Primary care system in Nepal includes sub-health posts located in rural and remote areas staffed by health workers and, in some cases, auxiliary health workers and provide basic preventive, promotive, and curative services.

- Health Posts: Serve as decentralized primary care units managed by health assistants or auxiliary nurse midwives and offer immunizations, maternal/child health, basic outpatient services, and health education.
- Primary Health Care Centers: Larger facilities serving multiple villages provide more comprehensive outpatient care, basic laboratory services, and minor procedures and are staffed by medical officers and nurses.
- Community Health Clinics: Function as referral points for Primary Health Care Centers and offer outpatient services, maternal health, family planning, and some diagnostic facilities.

These centers are funded by the government of Nepal through the Ministry of Health and Population, where budget allocations come from national treasury, local governments, and provincial health authorities, especially after federalization. Nepal Health Sector Program is another major funding and support program to strengthen primary health care infrastructure, workforce, and services. Moreover, donor support and international aid from organizations such as World Health Organization, United Nations International Children's Emergency Fund, and nongovernmental organizations support infrastructure, training, and health campaigns, supplementing government resources. Similarly, local governments are increasingly involved in funding and managing primary care facilities following Nepal's federal restructuring. Some services at health posts and clinics require minimal user fees, although key services such as maternal and child health are often free and efforts are ongoing to reduce or eliminate out-of-pocket expenses to improve access. Targeted screening strategies should be developed based on local risk factors, especially in underresourced health care settings. Screening should target high-risk groups, including the elderly, patients with hypertension, diabetes, family history of CKD, and other risk factors (Table 4). Screening tests should include serum creatinine-based eGFR, urine dipstick, urinalysis, and BP measurement. In resource-limited settings, screening for albuminuria may be sufficient. Early referral to nephrologists is crucial for optimal management of CKD, including specific therapies such as initiation of sodium-glucose cotransporter 2 inhibitors, nonsteroidal mineralocorticoid receptor antagonist although available cost and the compliance remains the concern, comorbidity management, and preparation for KRT. Referral criteria should include persistent and severe albuminuria, eGFR <30 ml/min per 1.73 m^2^, or a rapid decline in eGFR. To address the shortage of nephrologists, telemedicine, protocol-driven management, and shared care with PCPs could be considered.^12^

**Table 4:** CKD burden and primary care infrastructure in Nepal

Aspect	Current Status	Source
CKD prevalence (population-based study)	6.0%	Poudyal *et al.*^6^, *BMJ Open* 2022
Leading causes of CKD	Diabetes mellitus, hypertension, GN (children), environmental toxins	Poudyal *et al.*^6^, *BMJ Open* 2022; Jayasumana *et al.*^11^, 2017
Screening programs	Limited, mostly opportunistic	MoHP, 2023
Primary care structure	SHPs, HPs, PHCCs, and CHCs	Nepal Health Sector Strategy 2020–2025
CKD medication access	SGLT2i, nsMRA available but expensive, low use	Author observation, national formulary data
CKD care funding	Covered under health insurance, chronic illness subsidy, and UHC policies	MoHP Health Insurance Board, 2023

CHC, community health clinics; HP, health post; MoHP, Ministry of Health and Population; nsMRA, nonsteroidal mineralocorticoid receptor antagonist; PHCC, primary health care centers; SGLT2i, sodium-glucose cotransporter 2 inhibitors; SHP, sub-health post; UHC, universal health coverage.

## Dialysis

Although patients receive free hemodialysis twice a week for 4 hours per session under the government program, dialysis adequacy and quality measures are not regularly assessed. This is particularly concerning given the well-defined international guidelines and our rapidly growing dialysis population. To safeguard the safety of dialysis treatment, the government conducts routine inspections for water quality, infection control, and dialyzer reuse. Most prevalent patients on hemodialysis have an arterio-venous fistula (AVF) as their primary access, while most incident patients began hemodialysis with a temporary access, typically nontunneled internal jugular catheters which are later converted to AVF.^13^ A study representing approximately 10% of all hemodialysis patients in Nepal showed a very low incidence of AVF use (25%) at the initiation of hemodialysis which supports the fact that most patients are reluctant for AVF creation and starts hemodialysis with temporary access.^13^ Reluctance to undergo AVF creation is multifactorial and largely driven by late patient presentation, limited awareness, cultural concerns, restricted surgical access, financial barriers, and uncertainty about the long-term continuation of dialysis (Table 1). Many dialysis centers in rural areas do not have physician's onsite, leaving patients dependent on nurses for care during dialysis. In centers with nephrologists on duty, physicians typically do not examine patients during hemodialysis sessions unless there is a critical issue that requires their attention. Regarding PD, the uptake remains very limited. It is estimated that only 11% of patients with ESKD currently receive PD as continuous ambulatory PD primarily due to economic constraints and unsuitable home environments. Additional barriers include the high cost of PD cyclers as well as inadequate clinic staffing and training.

## Interventions and Dialysis Access Creation

With the exception of renal biopsy, very few nephrologists are involved in interventions due to the lack of training and heavy workload. Typically, the critical care team—intensivists or anesthesiologists—insert internal jugular hemodialysis catheters; cardiovascular surgeons are responsible for placing nearly all arteriovenous fistulas, while general surgeons perform peritoneal catheter insertions. In cases of emergency hemodialysis, a temporary femoral catheter is usually placed by either a physician or a dialysis nurse.

## Renal Transplant Service

As stated previously, the first successful solid organ transplantation in Nepal took place in 2008, following the enactment of the Human Body Transplantation Act in 1998. The transplantation program primarily focuses on living donor transplants, with strict eligibility requirement for organ donors, limiting donations to close relatives. However, owing to limited access to genetic testing, the well-being of living related donors at risk for inherited diseases cannot be fully ensured. Amendments to the Act in 2016 introduced organ criteria for brain death, enabled paired exchange, and expanded the scope of related donor eligibility. There are no cardiac-death donor programs at present. SDNTC performed the first brain-dead donor kidney transplant in 2016.^14^ Most transplantations take place in the capital city, with SDNTC, TUTH, and Bir Hospital serving as the main government centers, while private facilities such as Kist Medical College, Kathmandu Medical College, Grande International Hospital, Norvic International Hospital, and Nepal Mediciti Hospital also provide transplant services Table 3. Outside the capital, kidney transplants are now being performed by Pokhara Academy of Health Sciences, a government facility. As of June 2025, approximately 3090 kidney transplants have been performed in Nepal, with SDNTC and TUTH leading the effort.^15,16^

## Critical Care Nephrology

Critical care nephrologists play a vital role in taking care of patients admitted to intensive care unit team with kidney diseases, electrolyte/acid base disorders, and metabolic derangements, working closely other medical professionals to provide multidisciplinary care. In Nepal, there is a pressing need for enhanced training in critical care nephrology for general nephrologists to improve care quality and patient safety, explore novel treatment options, establish evidence-based protocols for conditions such as AKI, KRT, and lead or participate in clinical trials.^17^ Their expertise will directly affect patient outcomes.

## Pediatric Nephrology Service

Pediatric nephrology in Nepal poses significant challenges typical of underresourced settings, yet there have been notable advances in recent years. The burden of glomerular diseases remains high, with nephrotic syndrome being the most common indication for intervention in the form of renal biopsy in children. Minimal change disease and IgA nephropathy are most frequently diagnosed primary GN, while lupus nephritis is the leading secondary GN.^18,19^ Acute poststreptococcal GN continues to be a major cause of pediatric hospitalizations, particularly affecting children from economically disadvantaged backgrounds. Histopathologic diagnosis relies on light microscopy and direct immunofluorescence, electron microscopy, due to the lack of advanced diagnostic facilities sample has to be sent to neighbor country and as genetic studies is limited, reflecting broader infrastructural constraints and difficulties in diagnosing the rare genetic diseases.^20^

Diagnostic challenges are further compounded by limited access to serologic and complement testing in rural areas. Most cases are managed conservatively, but severe presentations requiring steroids, kidney biopsies, or KRT have to be referred to the capital city of Kathmandu. Despite these limitations, short-term outcomes are generally favorable, with low mortality even among those requiring advanced interventions.^21^

Kidney transplantation in children is available but remains rare, with only a small proportion of total transplants performed in pediatric patients. Living donor transplantation is the major form, predominantly mothers being a donor.^22^

## Future Directions

Indeed, significant progresses have been made in improving kidney care in Nepal over the years. However, substantial improvements are still needed, provided current challenges are effectively addressed. National-level advocacy is essential not only for promoting disease awareness and prevention but also for reducing the cost of care by leveraging economies of scale. In addition, an increase in insurance support for kidney disease-related costs is necessary to optimize patient outcomes in our population. Further investments in medical infrastructure outside major cities and home dialysis are essential to reduce disparities in care and improve access to health care services. Finally, developing a skilled nephrology workforce is another key priority. In an increasingly interconnected world, partnerships with more experienced global medical centers could help support nephrology training in Nepal. This will be particularly valuable for building human resource capacity and increasing the availability of well-trained future nephrologists.
